# Supplementary material for: Hyaluronic Acid-Coated Nanoliposomes as Delivery Systems for Fisetin: Stability, Membrane Fluidity, and Bioavailability
Source: Foods. 2024 Jul 29;13(15):2406. doi: 10.3390/foods13152406 (PMC11311619; doi:10.3390/foods13152406)
Supplement: Supplementary file 1 [file foods-13-02406-s001.zip › foods-3011872-supplementary.pdf]

Table S1. Effect of HA MW on liposome particle size, potential, and polydispersity index.

MW	Mean size (nm)	PDI	Zeta potential (mV)
0 Da	79.46 ± 0.06 c	0.265 ± 0.001 a	-2.65 ± 0.64 b
3000 Da	100.75 ± 0.64 b	0.284 ± 0.037 a	-1.70 ± 0.22 ab
35 kDa	100.25 ± 0.21 b	0.267 ± 0.016 a	-1.30 ± 0.43 a
90-100 kDa	100.55 ± 0.49 b	0.261 ± 0.008 a	-1.41 ± 0.26 a
150-250 kDa	101.10 ± 0.99 b	0.269 ± 0.005 a	-1.82 ± 0.66 ab
1000-1500 kDa	104.20 ± 1.56 a	0.299 ± 0.028 a	-2.09 ± 0.26 ab

Table S2. Effect of HA concentration on liposome particle size, potential, and polydispersity index.

HA concentration	Mean size (nm)	PDI	Zeta potential (mV)
0	82.10 ± 2.79 c	0.312 ± 0.042 a	-4.25 ± 3.46 a
0.1%	93.63 ± 4.06 a	0.336 ± 0.022 a	-3.96 ± 0.30 a
0.4%	87.24 ± 0.03 bc	0.308 ± 0.004 a	-3.05 ± 1.15 a
0.7%	94.75 ± 2.09 a	0.342 ± 0.017 a	-2.46 ± 0.12 a
1.5%	90.86 ± 0.54 ab	0.308 ± 0.037 a	-2.23 ± 0.72 a
